# Transcription factor myocyte enhancer factor 2D regulates interleukin-10 production in microglia to protect neuronal cells from inflammation-induced death

**DOI:** 10.1186/s12974-015-0258-z

**Published:** 2015-02-20

**Authors:** Shaosong Yang, Li Gao, Fangfang Lu, Bao Wang, Fei Gao, Gang Zhu, Zhibiao Cai, Juan Lai, Qian Yang

**Affiliations:** Department of Neurosurgery, Tangdu Hospital, The Fourth Military Medical University, Xi’an, 710038 China

**Keywords:** MEF2D, IL-10, Microglia, Neuroinflammation, Parkinson’s disease

## Abstract

**Background:**

Neuroinflammatory responses have been recognized as an important aspect in the pathogenesis of Parkinson’s disease (PD). Transcriptional regulation plays a critical role in the process of inflammation. Transcription factor myocyte enhancer factor 2D (MEF2D) is identified as a central factor in transmission of extracellular signals and activation of the genetic programs in response to a wide range of stimuli in several cell types, including neurons. But its presence and function in microglia have not been reported. We therefore investigated the effect of MEF2D in activated microglia on the progress of neuroinflammation and the survival of neurons.

**Methods:**

BV2 cells and primary cultured glial cells were stimulated with lipopolysaccharide (LPS). Samples from cells were examined for MEF2D expression, interleukin-10 (IL-10), and tumor necrosis factor alpha (TNF-α) by immunoblotting, quantitative real-time PCR (qPCR) or enzyme-linked immunosorbent assay (ELISA). The activity of MEF2D was examined by electrophoretic mobility shift assay (EMSA) and chromatin immunoprecipitation assay (ChIP). Recombinant lentivirus expressing shRNA specific to MEF2D was used to silence MEF2D expression in BV2 cells. The role of IL-10 transcriptionally induced by MEF2D on neuronal survival was assessed by anti-IL-10 neutralizing antibody. The survival of neurons was determined by 3-(4,5-dimethylthiazol-2-yl)-2,5-diphenyltetrazolium bromide (MTT) and terminal deoxynucleotidyl transferase-mediated dUTP nick end labeling (TUNEL) staining. Male C57bl/6 mice were used to establish an acute PD model. Brain sections and cell slides were tested by immunofluorescence.

**Results:**

We demonstrated that MEF2D was present in microglia. Activation of microglia was associated with an increase in MEF2D level and activity in response to different stimuli *in vivo* and *in vitro*. MEF2D bound to a MEF2 consensus site in the promoter region of *IL-10* gene and stimulated IL-10 transcription. Silencing MEF2D decreased the level of IL-10, increased the TNF-α mRNA, and promoted inflammation-induced cytotoxicity, consistent with the result of inhibiting IL-10 activity with an anti-IL-10 neutralizing antibody.

**Conclusions:**

Our study identifies MEF2D as a critical regulator of *IL-10* gene expression that negatively controls microglia inflammation response and prevents inflammation-mediated cytotoxicity.

**Electronic supplementary material:**

The online version of this article (doi:10.1186/s12974-015-0258-z) contains supplementary material, which is available to authorized users.

## Background

Parkinson’s disease (PD) is the second most common neurodegenerative disorder and characterized pathologically by the loss of pigmented dopaminergic (DA) neurons in the substantial nigra pars compacta (SNc) [1]. The precise reasons for the selective loss of SNc DA neurons are not entirely clear. Despite intensive researches on DA neurons, recent studies show that glial cells, especially microglia, may be involved in the progressive degeneration of DA neurons [2-4]. Studies of post-mortem brain tissue of PD patients show the presence of activated microglia in the affected brain regions, strongly supporting that neuroinflammatory process is associated with neuron loss [5]. Furthermore, the activated microglia has been shown to contribute to DA neuronal death not only by releasing pro-inflammation factors such as tumor necrosis factor alpha (TNF-α) and interleukin-1 beta (IL-1β) [6,7] but also by producing hyperoxides and peroxidases including reactive oxygen species (ROS) and NADPH oxidase [8-10].

As an important innate immunity in the central nervous system (CNS), modest activated microglia is necessary and beneficial for brain health. But controlling over vigorous immune responses is critical for preventing this reactivity from being over-activated and damaging the brain. A lot of such controlling mechanisms may contribute to the chronic pathogenesis in neurodegenerative diseases [11,12]. Interleukin-10 (IL-10) is an anti-inflammation cytokine with important roles in preventing inflammation [13]. IL-10 regulates exuberant immune response of microglia by inhibiting their release of pro-inflammatory mediators such as TNF-α and IL-1β [14] and increasing the release of anti-inflammatory mediators such as IL-1 receptor antagonist (IL-1ra) and soluble TNF-α receptors [15,16]. Given the critical role of IL-10 in regulating the course of microglia-mediated inflammatory response, elucidating how IL-10 is regulated may provide valuable information for understanding the pathogenesis of PD. One important aspect of controlling of IL-10 is the level of its gene transcription. The structure of *IL-10* gene promoter contains AT-rich putative transcription factor myocyte enhancer factor 2 (MEF2) binding site. Recent evidence from lymphocytes identifies *IL-10* gene as a potential MEF2 transcriptional target [17,18].

MEF2s, initially identified as a nuclear factor important for muscle cell differentiation [19], have four mammalian isoforms, MEF2, A to D. The N-terminus of MEF2 mediates dimerization and DNA binding, while the C-terminus of MEF2 functions as transcriptional activation domains. MEF2s have been found to play a central role in the activation of the genetic programs that regulate cell proliferation, differentiation, and apoptosis in increasing types of cells [20]. Our previous study demonstrates that MEF2D promotes the survival of DA neurons in the SNc under stress conditions. Negative regulation of MEF2D by toxic signals contributes to DA neuronal death [21].

In spite of the studies of MEF2D in neurons, its function and regulation in microglia are entirely unknown. In the present study, we examined the function of MEF2D in activated microglia. Our data showed that the expression and activity of MEF2D were significantly induced in activated microglia. MEF2D regulated the expression for IL-10 in microglia. Silencing MEF2D expression led to a decrease in IL-10 mRNA and protein. This contributed to an increase in inflammation-induced and microglia-mediated toxicity to DA neuronal cells. These results establish a direct link between MEF2D and IL-10 activity in microglia-mediated inflammatory response, suggesting that MEF2D may play a critical role in preventing over-exuberant immune responses and protecting neurons from microglia-mediated neurotoxicity in PD.

## Methods

### Animal and tissue preparations

C57bl/6 male mice (25 ~ 30 g), purchased from the Experimental Animal Center of the Fourth Military Medical University, were used according to the Guidelines for Animal Care and Use of the Fourth Military Medical University (Xi’an, People’s Republic of China). All efforts were made to minimize animal suffering and to reduce the number of animals used. Mice received four intraperitoneal (i.p.) injections of 20 mg/kg free base 1-methyl-4-phenyl-1,2,3,6-tetrahydropyridine (MPTP) (Sigma-Aldrich, St. Louis, MO, USA) at 2-h intervals. Control mice were injected with phosphate buffer solution (PBS) alone at the same frequency. At 1 day, animals were anesthetized (10% chloralhydrate, i.p.) and transcardially perfused with PBS. The brains were fixed with cold 4% paraformaldehyde. Serial brain sections (30 μm thick) containing the SNc were collected for further analysis.

### Cell culture and treatment

BV2 cells were cultured in Dulbecco’s modified Eagle’s medium/F12 (DMEM/F12 containing 2.8 mM L-glutamine, 15 mM HEPES) (Gibco, Grand Island, NY, USA) supplemented with 5% fetal bovine serum (FBS) (Gibco), and incubated with 5% CO_2_ at 37°C.

Primary mixed glial cell cultures were derived from post-natal days 0 to 3 (P0-P3) Sprague-Dawley rat brains. Shortly, the whole brains were dissociated in trypsin without EDTA for 10 min at 37°C and then were cultured in DMEM/F12 supplemented with 10% FBS. Mixed glial cell cultures, which were cultured in poly-D-lysine-coated (0.1 mg/ml) (Sigma-Aldrich) T75 flasks and on cell slides, were incubated at 37°C and 5% CO_2_ for 13 to 16 days. When the cells in T75 flasks became confluent, the flasks were shaken at 250 rpm/min for 2 h to detach microglia. The microglia after purification were cultured in DMEM/F12 with 10% FBS for 3 days before their use for the following experiments.

SN4741, a mouse embryonic substantial nigra-derived cell line [22], was cultured at 33°C with 5% CO_2_ in RF medium (DMEM with 10% FBS, 1% D-glucose, 1% penicillin-streptomycin, and 140 mM L-glutamine). Experiments were usually done when cells reached 50% to 60% confluence.

When reaching 60% to 70% confluence, the BV2 cells and primary microglia cells were exposed to lipopolysaccharide (LPS, 1.0 μg/ml) (Sigma-Aldrich) for a designed time point, respectively. Then, the cells were enriched for the following experiments.

For neutralizing antibody experiments, BV2 cells were treated with 1.0 μg/ml LPS in the presence or absence of anti-IL-10 neutralizing antibody (1.0 μg/ml, Abcam, Cambridge, UK) for 24 h.

### Lentivirus infection

Recombinant lentivirus vector expressing shRNA-MEF2D to silence MEF2D gene expression was obtained commercially from Hanbio, Shanghai, China. BV2 cells were seeded in a six-well plate and were infected with lentivirus in 1 ml medium for 4 h. The medium was added to 2 ml, and cells were continuously infected for another 24 h. After being replaced with fresh medium, the cells were cultured and used for studies.

### Immunofluorescence

Mixed glia cell slides and brain sections were fixed with 4% formaldehyde solution, incubated with 0.1% Triton X-100 for 30 min, and blocked with 5% bovine serum albumin (BSA) (Sigma-Aldrich) in PBS for 30 min. Samples were incubated at 4°C overnight with primary anti-Iba-1 (1:50; Abcam, Cambridge, UK) combined with anti-MEF2D (1:200; BD Transduction Laboratories, San Jose, CA, USA); then they were washed three times with PBS and incubated with secondary antibody conjugated with FITC in room temperature for 2 h. Samples were counterstained with DAPI (1:500; Sigma) for 10 min and photographed using a confocal microscope (C2 Si, Nikon, Minato, Japan).

### Immunoblotting

Cell protein was harvested with ice-cold lysis buffer containing protease inhibitors. Protein was separated by sodium dodecyl sulfate polyacrylamide gel electrophoresis (SDS-PAGE), and then the separated protein was transferred onto polyvinylidene fluoride (PVDF) membranes (Millipore Corporation, Bedford, MA, USA). After 2-h blocking with 5% fat-extracted milk at room temperature, the membranes were incubated overnight at 4°C with primary antibodies against MEF2D (1:3,000; BD) and β-actin (1:4,000; Abcam). Then the membranes were washed with TBST three times and then were treated with horseradish peroxidase-conjugated secondary antibody for 2 h at room temperature. Protein bands were visualized by chemiluminescence detection.

### Quantitative real-time PCR

Total RNA was extracted from each sample according to the manufacturer’s protocol for TRIzol reagent (Invitrogen, Paisley, Scotland, UK). Complementary DNA (cDNA) was generated from total RNA using random primer and MMLV reverse transcriptase (Invitrogen), using a final volume of 20 μl. Quantitative real-time PCR (qPCR) analysis was performed in triplicate using QuantiFast™ SYBR® Green PCR Kit (Qiagen, Hilden, Germany). All gene-specific mRNA expression values were compared to β-actin mRNA levels as a standard. The primer sequences for each gene are listed as follows:

- MEF2D forward, 5′-CGTTGGGAATGGCTATGTC-3′;
- MEF2D reverse, 5′-GAGGCCCTGGCTGAGTAA-3′;
- IL-10 forward, 5′-GCTCTTACTGACTGGCATGAG-3′;
- IL-10 reverse, 5′-CGCAGCTCTAGGAGCATGTG-3′;
- β-actin forward, 5′-AAGGACTCCTATAGTGGGTGACGA-3′;
- β-actin reverse, 5′-ATCTTCTCCATGTCGTCCCAGTTG-3′.

### Chromatin immunoprecipitation assay

Chromatin immunoprecipitation assay (ChIP) was done following a procedure provided by a ChIP Assay Kit (Millipore). The product of DNA was analyzed by using qPCR and semiquantitative PCR with *IL-10* gene promoter specific primers as follows:

- forward, 5′-CTGTCTGCCTCAGGAAAT-3′;
- reverse, 5′-CTAAAGAACTGGTCGGAAT-3′.

### Electrophoretic mobility shift assay

Electrophoretic mobility shift assay (EMSA) was performed as described [23]. The Binding reactions were incubated with MEF2 specific probe or mutant probe, and for super shift assay, reactions were incubated with MEF2D antibody for 1 h at 4°C after incubation with labeled probe. The sequences of the probes used for EMSA are as follows:

Wild type:

- 5′-TCGACGGGCTATTTTTAGGGCC-3′/
- 3′-AGCTGCCCGATAAAAATCCCGG-5′;

Mutant:

- 5′-TCGACGGGCGATTTTTCGGGCCG-3′/ 3′-AGCTGCCCGCTAAAAAGCCCGGC-5′

### Enzyme-linked immunosorbent assay

Detection of IL-10 in the supernatant of treated and untreated BV2 cells cultures was determined with a mouse IL-10 enzyme-linked immunosorbent assay (ELISA) kit (R&D Systems, Wiesbaden, Germany) according to manufacturer procedures, and results were raised as picogram per milliliter.

### MTT assay

Cells were seeded in 96-well plates (5 × 10^3^ cells/well) and incubated at 37°C. After treatment, 20 μl of 3-(4,5-dimethylthiazol-2-yl)-2,5-diphenyltetrazolium bromide (MTT) (5 mg/ml) (Millipore) were added to each well, and the plate was incubated for 4 h. Then, after removing supernatant, 150 μl of dimethylsulfoxide (DMSO) (Millipore) was added to each well and mixed thoroughly for 10 min. The optical density (OD) was measured at 490 nm.

### TUNEL staining

For terminal deoxynucleotidyl transferase-mediated dUTP nick end labeling (TUNEL) staining, FragEL™ DNA Fragmentation Detection Kit (Millipore) was used according to manufacturer’s instructions.

### Statistical analyses

Data were expressed as mean ± standard error of the mean (SEM) from at least three independent experiments. Data were analyzed by either one-way ANOVA or two-way ANOVA as appropriate. Statistical analyses were carried out using SPSS 19.0. A value of *P* < 0.05 was considered statistically significant.

## Results

### Induction of MEF2D expression during microglia activation

**Figure 1:**
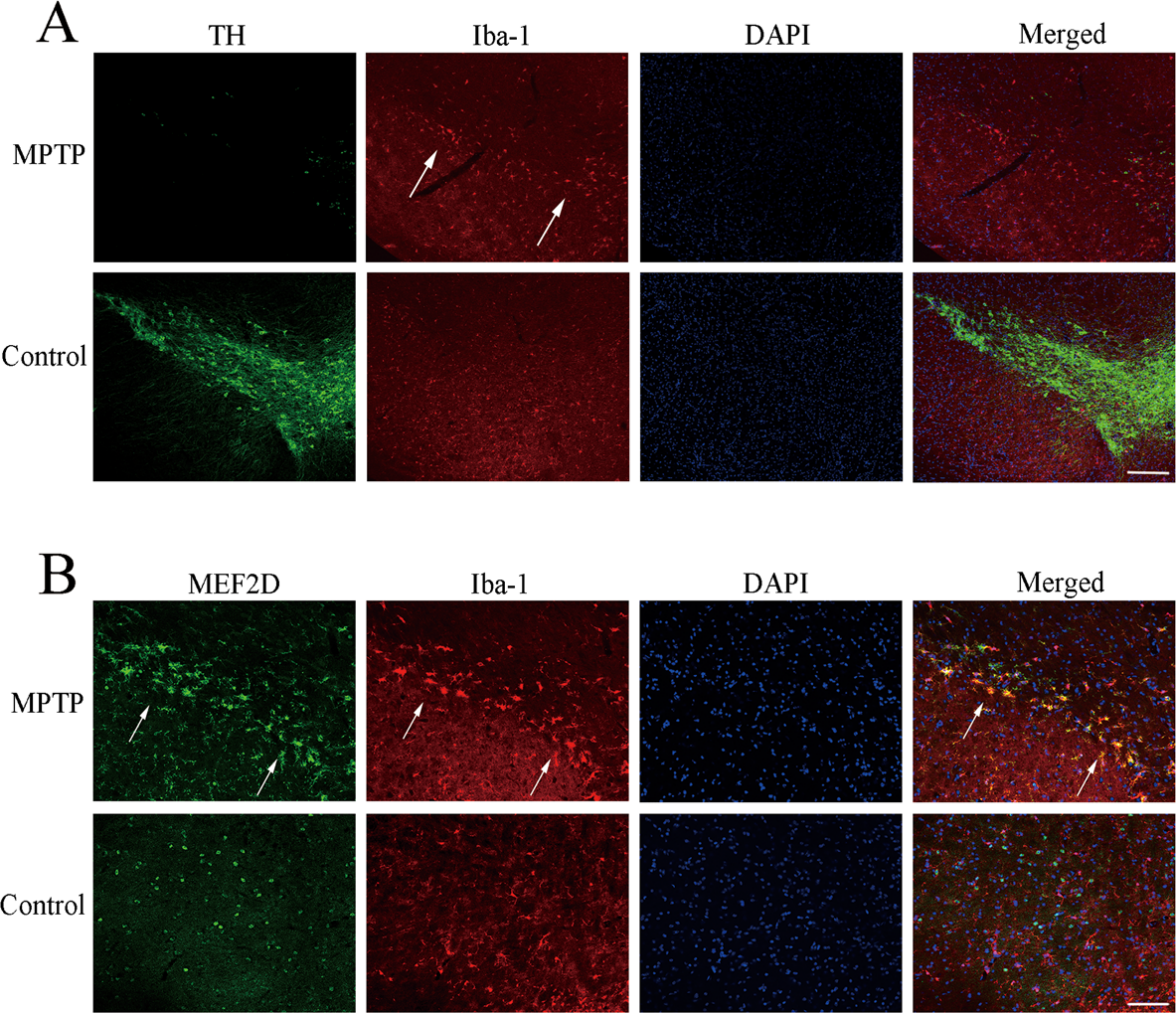
**Induction of MEF2D expression in the activated microglia. (A)** Loss of TH signals in MPTP-treated mouse brain. SNc brain sections from mice injected with MPTP were stained with the antibodies indicated by immunofluorescence (TH, green; Iba-1, red; bar = 200 μm; and *n* = 3). **(B)** Expression of MEF2D in activated microglia (MEF2D, green; Iba-1, red; bar = 100 μm; and *n* = 3).

Previous studies show that MEF2s participate in the regulation of immune responses in T and B lymphocytes [24,25]. That prompted us to test whether MEF2D might be involved in inflammatory response in the processes of PD. For this, we used neurotoxin MPTP-induced loss of SNc DA neurons, a widely used model of PD. After 24 h of MPTP administration, we examined the effects of this toxin on tyrosine hydroxylase (TH)-positive DA neurons in SNc. The acute MPTP exposure clearly caused a loss of immunohistochemical signal for TH in SNc. We stained the same brain sections for Iba-1, a marker for activated microglia, and found that MPTP induced a significant increase in Iba-1 signal (Figure 1A). We then stained the brain section with a monoclonal anti-MEF2D antibody. This antibody recognizes MEF2D as a single band by immunoblotting and has been used successfully in immunostaining of brain tissues [26]. Immunofluorescence analysis showed that MEF2D expression was clearly increased in thick bundles around swollen somata microglia in SNc region after MPTP injection. Moreover, the high levels of MEF2D expression were colocalized with Iba-1 (Figure 1B). These data demonstrate that MEF2D level increases with the activation of microglia in MPTP-PD animal model.

### Induction of expression and activity of MEF2D in BV2 cells

**Figure 2:**
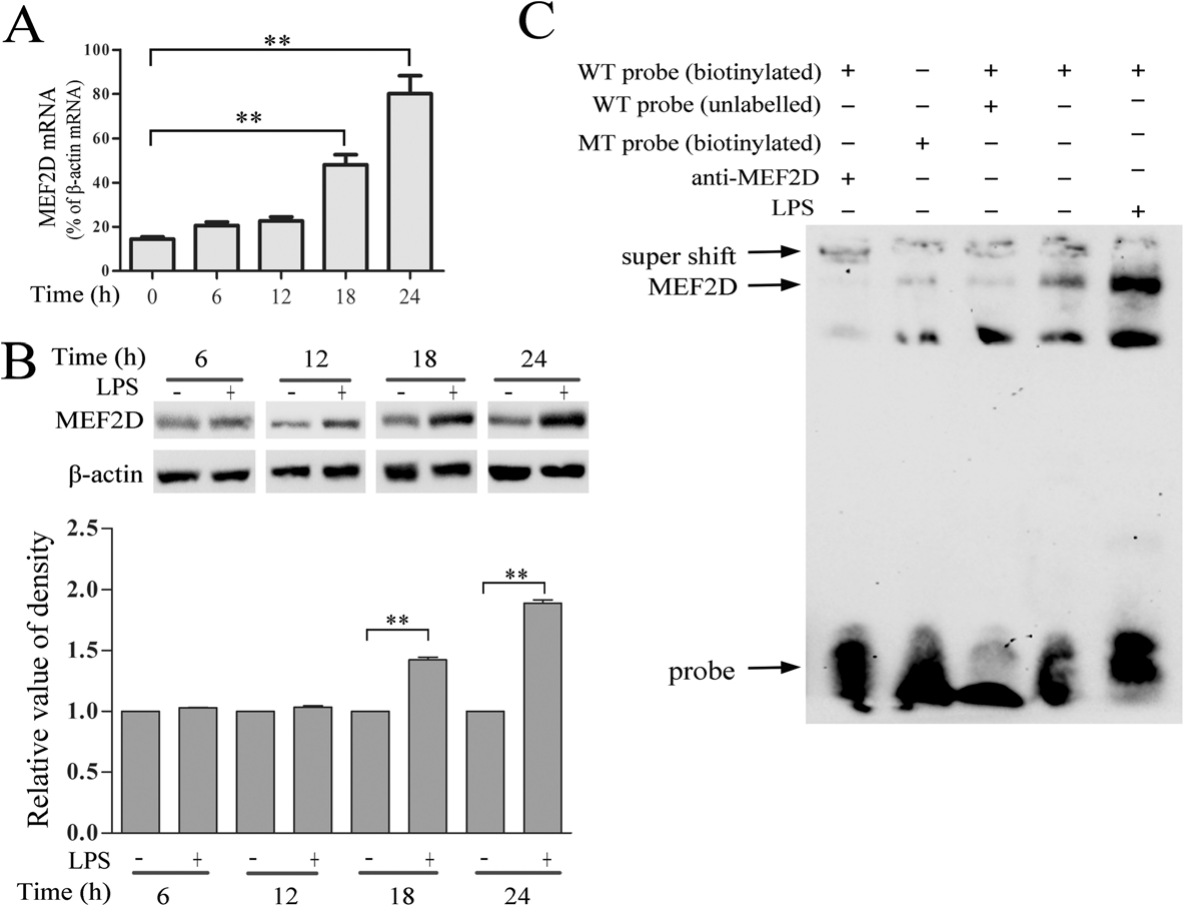
**LPS-induced increase in expression and activity of MEF2D in BV2 cells. (A)** LPS-induced increase in MEF2D mRNA expression in BV2 cells. BV2 cells were exposed to 1.0 μg/ml LPS for 6, 12, 18, or 24 h, and the mRNA levels of MEF2D were quantified by qPCR and normalized to β-actin mRNA level as described in ‘Methods’. Data from three independent experiments were expressed as the mean ± SEM and analyzed by one-way ANOVA (**P < 0.01). **(B)** LPS-induced increase in MEF2D protein in BV2 cells. BV2 cells were treated for the indicated time as described above and immunoblotted for MEF2D. (**B** bottom graph) Relative quantification of MEF2D. Data were expressed as mean ± SEM from at least three independent experiments and analyzed by one-way ANOVA (**P < 0.01). **(C)** LPS-induced increase in MEF2D activity. The whole cell extracts were prepared from BV2 cells treated with or without LPS for stimulating 24 h and analyzed by EMSA. Arrow indicates the position of MEF2D-probe complex (unlabelled probe: a 100-fold excess of unlabelled probe was added in the reaction; MT probe: probe with MEF2 binding site mutated).

To further confirm MEF2D expression and investigate its role in microglia, we performed experiments in BV2 cells, a mouse microglia cell line. We first tested MEF2D mRNA expression by qPCR under basal condition. There was a low but detectable MEF2D mRNA in BV2 cells. The level of MEF2D transcript was gradually increased following LPS treatment, reaching a significantly higher level at 18 and 24 h after LPS treatment (Figure 2A). In correlation with the change of MEF2D mRNA, the level of MEF2D protein was also increased gradually following LPS treatment. The level of MEF2D protein gradually increased 12 h after LPS exposure and reached a much higher level 24 h post-treatment (Figure 2B). To examine this fact in different cell models, we cultured mixed glial cells or purified primary microglia and treated the cells with LPS. Our analysis showed that LPS treatment significantly increased the levels of MEF2D in both mixed glial and primary microglia cells by immunofluorescence or immunoblotting, respectively (Additional file 1: Figure S1A and B). To determine if the change in MEF2D level correlates with its activity, we performed EMSA to assess MEF2D DNA binding capacity. The whole cell extract was prepared from BV2 cells after 24 h of LPS treatment and examined for the binding to labeled DNA probe containing MEF2 binding consensus site. Control lysate showed a detectable formation of protein and probe complex (Figure 2C). Mutation of the MEF2 binding site in the DNA nearly abolished the formation of the complex. Addition of anti-MEF2D to the reaction super shifted the formation of the complex, indicating that MEF2D was a major protein component in the complex (Figure 2C). This formation of MEF2D and probe complex was significantly inducted in lysates after LPS treatment, suggesting that increased MEF2D activity accompanies LPS-induced activation of BV2 cells.

### Identification of MEF2D target DNA in microglia

**Figure 3:**
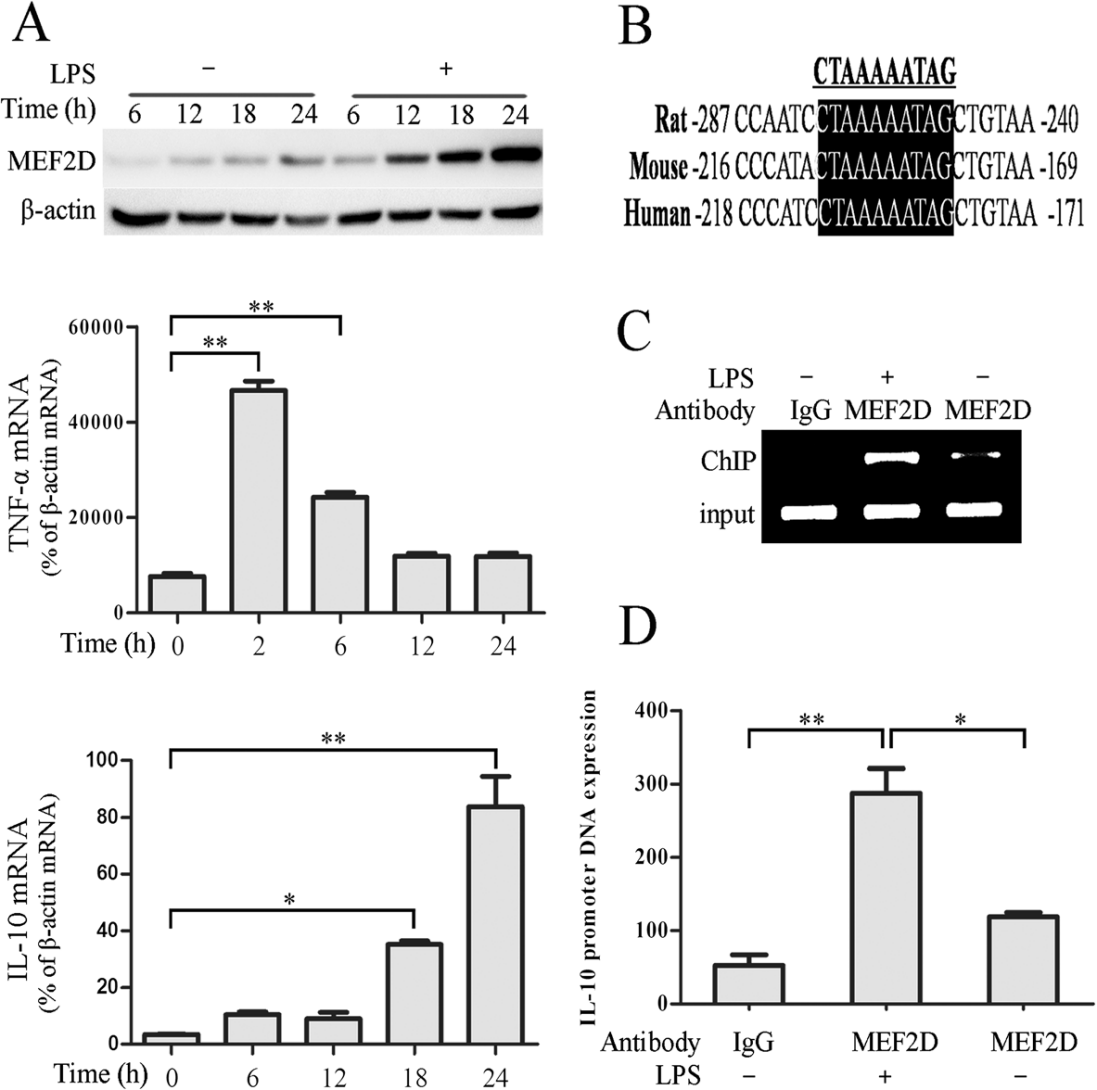
**Regulation of IL-10 gene expression by MEF2D. (A)** LPS-induced time-dependent changes of MEF2D protein and cytokines in BV2 cells. BV2 cells treated with LPS as described in Figure 2A were analyzed by immunoblotting for MEF2D and by qPCR for TNF-α and IL-10 mRNA levels. Data from three independent experiments were expressed as the mean ± SEM and analyzed by one-way ANOVA (*P < 0.05; **P < 0.01). **(B)** Identification of a conserved MEF2 binding site in the *IL-10* gene promoter of different species. The underlined sequence indicates the MEF2 binding site. Black-shaded areas show the conversed MEF2 binding site in the *IL-10* gene promoter of different species. **(C, D)** Binding of MEF2D to the *IL-10* gene promoter region containing the conserved site in BV2 cells. BV2 cells treated with or without LPS for 24 h were analyzed by ChIP. The result was showed by standard endpoint PCR **(C)** and qPCR **(D)**. Data from three independent experiments were expressed as the mean ± SEM and analyzed by one-way ANOVA (*P < 0.05; **P < 0.01).

Our above data showed that MEF2D expression was induced by LPS relatively late, suggesting that MEF2D may function in activated microglia rather than in the early activating stage. To test this, we performed time course studies to establish the pattern of MEF2D protein following LPS treatment (Figure 3A, top graph) and then assessed the mRNA levels of pro-inflammatory factor TNF-α and anti-inflammatory factor IL-10 following LPS. Our data showed that the level of TNF-α mRNA was induced early and reached its peak 2 h after LPS (Figure 3A, middle graph). In contrast, the level of IL-10 mRNA was induced more gradually and showed significant increase at 18 h after LPS treatment (Figure 3A, bottom graph). Thus, the pattern of IL-10 mRNA response matched well with that of MEF2D protein and prompted us to search for possible MEF2 regulatory target in *IL-10* gene promoter. Analyzing *IL-10* gene promoter sequence reveals the presence of a putative MEF2 binding site (5′-CC[A/t][t/a]AAATAG-3′) [27], which was conserved among several species examined (Figure 3B). To test whether MEF2D binds to this putative MEF2 site in *IL-10* gene, we carried out ChIP assay. Our ChIP analysis showed that MEF2D bound specifically to a region within *IL-10* gene promoter that contains the putative site in BV2 cells by either end product PCR (Figure 3C) or qPCR (Figure 3D). This binding was significantly induced in response to LPS treatment.

### Transcriptional regulation of *IL-10* gene by MEF2D

**Figure 4:**
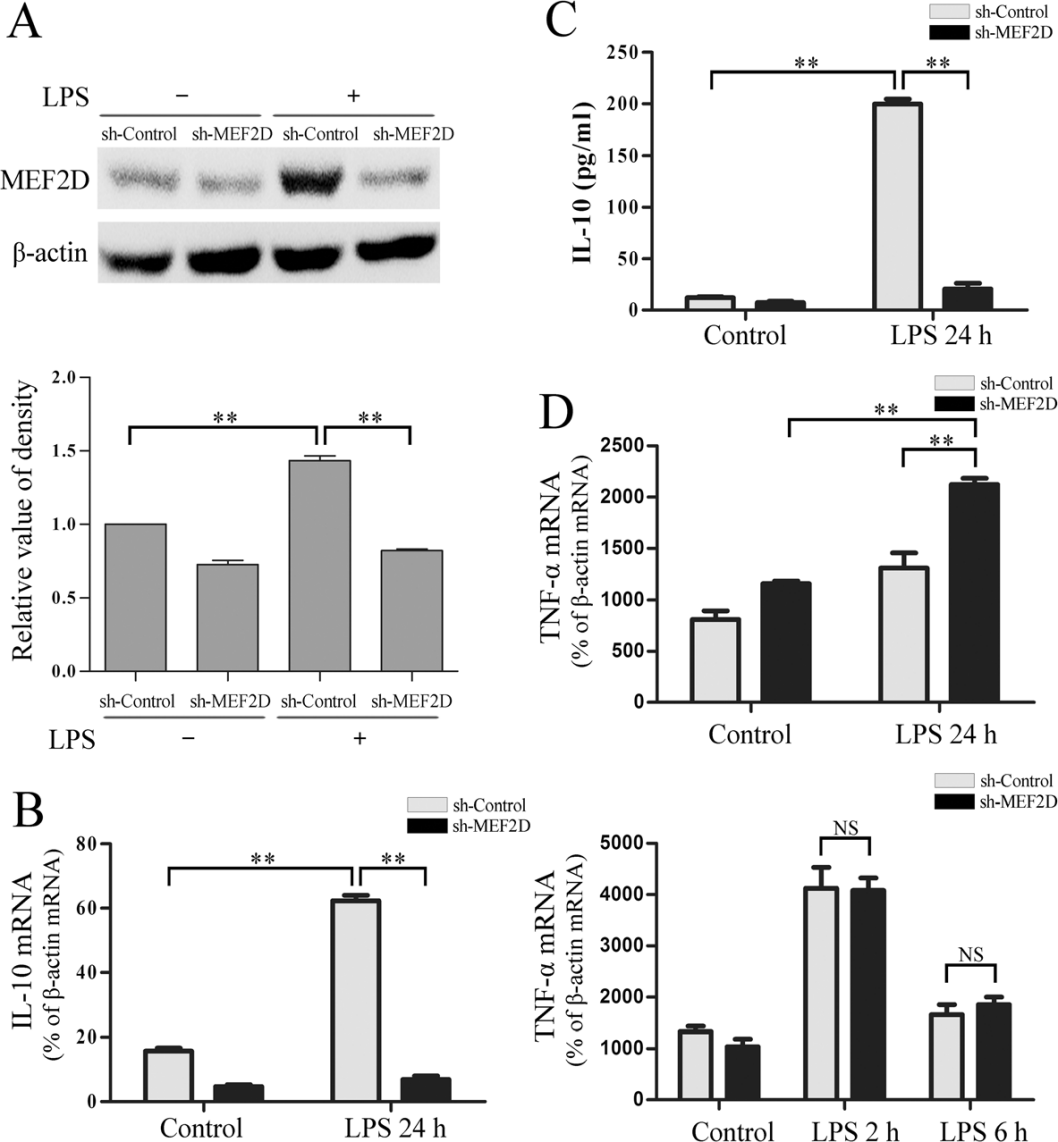
**Effect of knocking-down MEF2D on IL-10 expression. (A)** BV2 cells were infected with recombinant lentivirus expressing either control shRNA or shRNA to MEF2D (sh-MEF2D) and then treated with LPS for 24 h. The effectiveness of sh-RNA MEF2D was examined by immunoblotting. (**A** bottom graph) Relative quantification of MEF2D. Data from three independent experiments were expressed as the mean ± SEM and analyzed by one-way ANOVA (**P < 0.01). **(B)** The effect of sh-MEF2D on IL-10 mRNA in BV2 cells. BV2 cells infected with sh-MEF2D lentivirus were treated with LPS and analyzed for IL-10 mRNA by qPCR. Data from three independent experiments were expressed as the mean ± SEM and analyzed by two-way ANOVA (**P < 0.01). **(C)** The effect of sh-MEF2D on the level of secreted IL-10. Culture media from BV2 cells treated as descried under **(B)** were harvested for ELISA. Data from three independent experiments were expressed as the mean ± SEM and analyzed by two-way ANOVA (**P < 0.01). **(D)** The effect of sh-MEF2D on TNF-α mRNA in BV2 cells. BV2 cells treated as described under **(B)** and exposed to LPS for different times were analyzed for TNF-α mRNA by qPCR. Data from three independent experiments were expressed as the mean ± SEM and analyzed by two-way ANOVA (**P < 0.01).

To assess the role of MEF2D in *IL-10* gene transcription, we infected BV2 cells with recombinant lentivirus expressing shRNA specific to MEF2D (sh-MEF2D). Immunoblotting analysis showed that this approach effectively reduced LPS-induced increase in the level of MEF2D protein (Figure 4A). To test the effect of silencing MEF2D expression, we first analyzed the IL-10 mRNA level with qPCR after LPS treatment. The results showed that silencing MEF2D expression significantly reduced the level of IL-10 transcript in response to LPS (Figure 4B). Similarly, reducing MEF2D level also significantly attenuated the LPS-induced increase of IL-10 cytokine in culture media determined by ELISA (Figure 4C). As an anti-inflammatory cytokine, IL-10 plays a critical role in preventing inflammatory and autoimmune pathologies. One of protective mechanisms of IL-10 is through inhibiting the production of the proinflammatory cytokines such as TNF-α. Analysis of TNF-α mRNA by qPCR showed that silencing MEF2D also led to a significant increase in its level 24 h after LPS treatment (Figure 4D, top graph). In contrast, silencing MEF2D had little effect on TNF-α mRNA level at either 2 or 6 h of LPS treatment (Figure 4D, bottom graph). Together, these data suggest that MEF2D is required for *IL-10* gene transcription in the activated microglia. Silencing MEF2D-mediated IL-10 expression increases the TNF-α level during late stage in response to LPS.

### The protective effect of MEF2D-IL-10 on the death of TH^+^ neuronal cells

**Figure 5:**
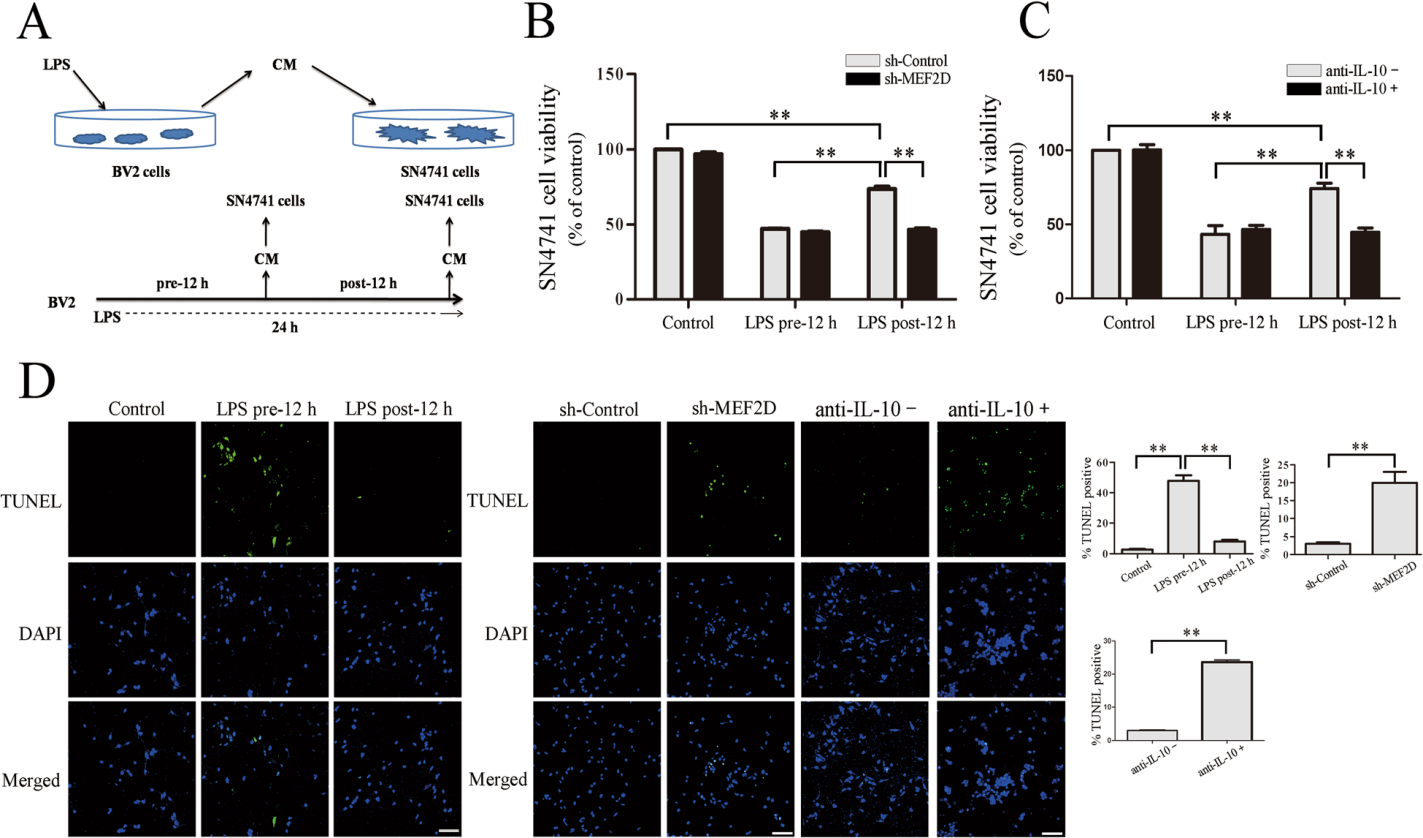
**The effect of knocking-down MEF2D on microglia-mediated cytotoxicity. (A)** Experiment paradigm for the collection of conditioned media (CM). Media collected from BV2 cells treated with LPS for 12 h were labeled as pre-12 h CM. After the first 12 h, cells were washed and placed in fresh media for another 12 h (post-12 h CM). CM was then added to SN4741 cells for 48 h. **(B)** The effect of silencing MEF2D on CM-mediated toxicity. CM was collected from BV2 cells infected with either control (sh-Control) or sh-MEF2D lentivirus (sh-MEF2D). The viability of SN4741 cells was determined by MTT. Data from three independent experiments were expressed as the mean ± SEM and analyzed by two-way ANOVA (**P < 0.01). **(C)** The effect of neutralizing IL-10 on CM-mediated toxicity. CM was collected from BV2 cells with (anti-IL-10+) or without (anti-IL-10−) anti-IL-10 neutralizing antibody. The viability of SN4741 cells was determined by MTT. Data from three independent experiments were expressed as the mean ± SEM and analyzed by two-way ANOVA (**P < 0.01). **(D)** The effect of silencing MEF2D or neutralizing IL-10 on CM-induced toxicity. For **(D)** left panel, the effect of CM from BV2 cells treated with LPS on SN4741 viability was assessed by TUNEL staining. For **(D)** middle panel, the effect of post-12 h CM collected from BV2 cells treated as in **(B)** or **(C)** were assessed by TUNEL assay in SN4741 cells. For **(D)** right panel, the quantification of the percentages of TUNEL-positive population. Data from three independent experiments were expressed as the mean ± SEM and analyzed by one-way ANOVA (**P < 0.01).

To test the effect of MEF2D-IL-10 on the survival of neurons, we designed a media transfer assay. For this experiment, we treated BV2 cells with LPS for 12 h, changed media, continued LPS treatment for another 12 h, collected conditioned media (CM), transferred CM to cultured DA neuronal line SN4741 cells, and measured neurotoxicity by MTT assay (Figure 5A). This analysis showed that CM collected from BV2 cells at the second 12 h was less toxic to SN4741 cells. Importantly, silencing MEF2D greatly enhanced the cytotoxicity of CM compared to control (Figure 5B). To confirm the effect of MEF2D-IL-10 on cellular survival and death, we co-treated cells with LPS and anti-IL-10 neutralizing antibody. Consistent with the effect of shRNA-MEF2D, blocking IL-10 clearly reduced the survival of SN4741 cells exposed to post-12 h CM by MTT assay (Figure 5C). TUNEL assay showed that pre-12 h CM caused a significant increase in the number of TUNEL-positive cells than post-12 h CM (Figure 5D, left panel). Silencing MEF2D expression and neutralizing IL-10 both significantly increased the toxicity of post-12 h CM (Figure 5D, middle panel). Thus, decreasing MEF2D alters the profile of cytokine and prolongs the cytotoxic effect of LPS.

## Discussion

Transcription factor MEF2s play important roles in neuronal development, synaptic plasticity, and survival [28-30]. Our previous studies show regulation of MEF2D activity by autophagy pathway is related to DA neuronal homeostasis and viability under stress conditions related to PD [23]. A few studies have reported a link between MEF2s and immune system. For example, MEF2s have been found to participate in immune responses of T and B lymphocytes. In addition, MEF2C level is found to increase in the activated microglia around the hippocampus after ischemia [31-33]. In the current study, we provide the first evidence demonstrating the presence of MEF2D in microglia and its function in microglia-mediated inflammation response. Our data indicate that in quiescent microglia, the levels of both MEF2D mRNA and protein are low. Stimulation by neurotoxin or LPS significantly induces MEF2D level and activity. Further, we demonstrate that the increase in MEF2D level is necessary for the transcriptional activation of *IL-10* gene expression and for damping the level of pro-inflammatory cytokines such as TNF-α. Thus, our data establish MEF2D as a critical negative regulator of neuroinflammation.

Our previous findings indicate that MEF2D functions in the nucleus and mitochondria in SNc DA neurons to promote neuronal survival [23,27]. Our current data have revealed that MEF2D-mediated regulation of IL-10 in microglia protects a cell line derived from DA neuronal progenitor against the cytotoxicity exerted by prolonged activation of microglia. Together, they indicate control of the over-activation of microglia as a novel mechanism by which MEF2D protects cells from inflammation-induced toxicity. It is important to investigate the temporal change of MEF2D in microglia in PD and determine if loss of MEF2D function in microglia may contribute inflammation-related injury in the pathogenic process of the disease.

Our data indicate that MEF2D mRNA level is robustly induced in microglia in response to LPS. The precise mechanisms underlying this induction remain unknown. It may involve either activation of *mef2d* gene transcription or increase in MEF2D mRNA stability. Robust increase in MEF2D transcript has been reported during hepatic stellate cells activation, a stimulation analogous to LPS-induced microglial response [34]. Since it is currently unclear how *mef2d* gene promoter is regulated, further studies are needed to clarify the regulatory mechanism(s) involved.

As an anti-inflammatory cytokine, IL-10 plays a critical role in controlling the intensity of inflammatory response by inhibiting production of various pro-inflammatory cytokines such as IL-1, IL-6, and TNF-α [35,36]. Expression of *IL-10* gene is highly inducible, which represents a major mechanism of regulation. Several transcription factors have been implicated in IL-10 regulation including C/EBPβ, SP1, NF-κB, and CREB under various conditions [37-40]. But how IL-10 expression is regulated in microglia has not been elucidated. Our data show that the kinetics of MEF2D expression and activity parallels closely with the induction of IL-10. MEF2D binds directly to a region in *IL-10* gene promoter that contains a well-conserved MEF2D binding site. Moreover, MEF2D activity is required for LPS-induced IL-10 mRNA expression. Together, our findings provide strong evidence supporting MEF2D as a primary transcription factor that directly regulates *IL-10* gene transcription in microglia.

## Conclusions

Our findings extend the cellular roles of MEF2D from neurons to microglia and further expand the mechanisms by which MEF2D regulates neuronal survival. Since communication between microglia and neurons has been increasingly being recognized to be important for various neuronal processes, our findings raise the question of whether MEF2D may play other roles in addition to regulation of inflammation in microglia and therefore is intricately involved in modulating microglia-neuron homeostasis. Overall, the beneficial role played by MEF2D in modulating microglia immune response supports the targeting MEF2D as a therapeutic strategy for reducing unwanted neuroinflammation.

## Additional file

Additional file 1: Figure S1. Induction of MEF2D expression by LPS in mixed glial cells and purified primary microglia. (A) Mixed glial cells from rat were treated with 1.0 μg/ml LPS for the indicated time and analyzed by immunofluorescence (bar = 25 μm). (B) Purified primary microglia was analyzed by immunoblotting. Bottom graph shows the quantification of MEF2D. Data from three independent experiments were expressed as mean ± SEM and analyzed by one-way ANOVA (***P* < 0.01).

## Abbreviations

BSA: bull serum albumin
ChIP: chromatin immunoprecipitation assay
CM: conditioned media
CNS: central nervous system
DA: dopaminergic
DMEM/F12: Dulbecco’s modified Eagle’s medium/F12
DMSO: dimethylsulfoxide
ELISA: enzyme-linked immunosorbent assay
EMSA: electrophoretic mobility shift assay
FBS: fetal bovine serum
IL-10: interleukin-10
IL-1ra: IL-1 receptor antagonist
IL-1β: interleukin-1 beta
LPS: lipopolysaccharide
MEF2D: myocyte enhancer factor 2D
MPTP: 1-methyl-4-phenyl-1,2,3,6-tetrahydropyridine
MTT: 3-(4,5-dimethylthiazol-2-yl)-2,5-diphenyltetrazolium bromide
OD: optical density
PBS: phosphate buffer solution
PD: Parkinson’s disease
PVDF: polyvinylidene fluoride
qPCR: quantitative real-time PCR
ROS: reactive oxygen species
SDS-PAGE: sodium dodecyl sulfate polyacrylamide gel electrophoresis
SNc: nigra pars compacta
TNF-α: tumor necrosis factor alpha
TUNEL: terminal deoxynucleotidyl transferase-mediated dUTP nick end labeling
