# Supplementary figures and images for: Transcription factor myocyte enhancer factor 2D regulates interleukin-10 production in microglia to protect neuronal cells from inflammation-induced death
Source: J Neuroinflammation. 2015 Feb 20;12:33. doi: 10.1186/s12974-015-0258-z (PMC4339472; doi:10.1186/s12974-015-0258-z)

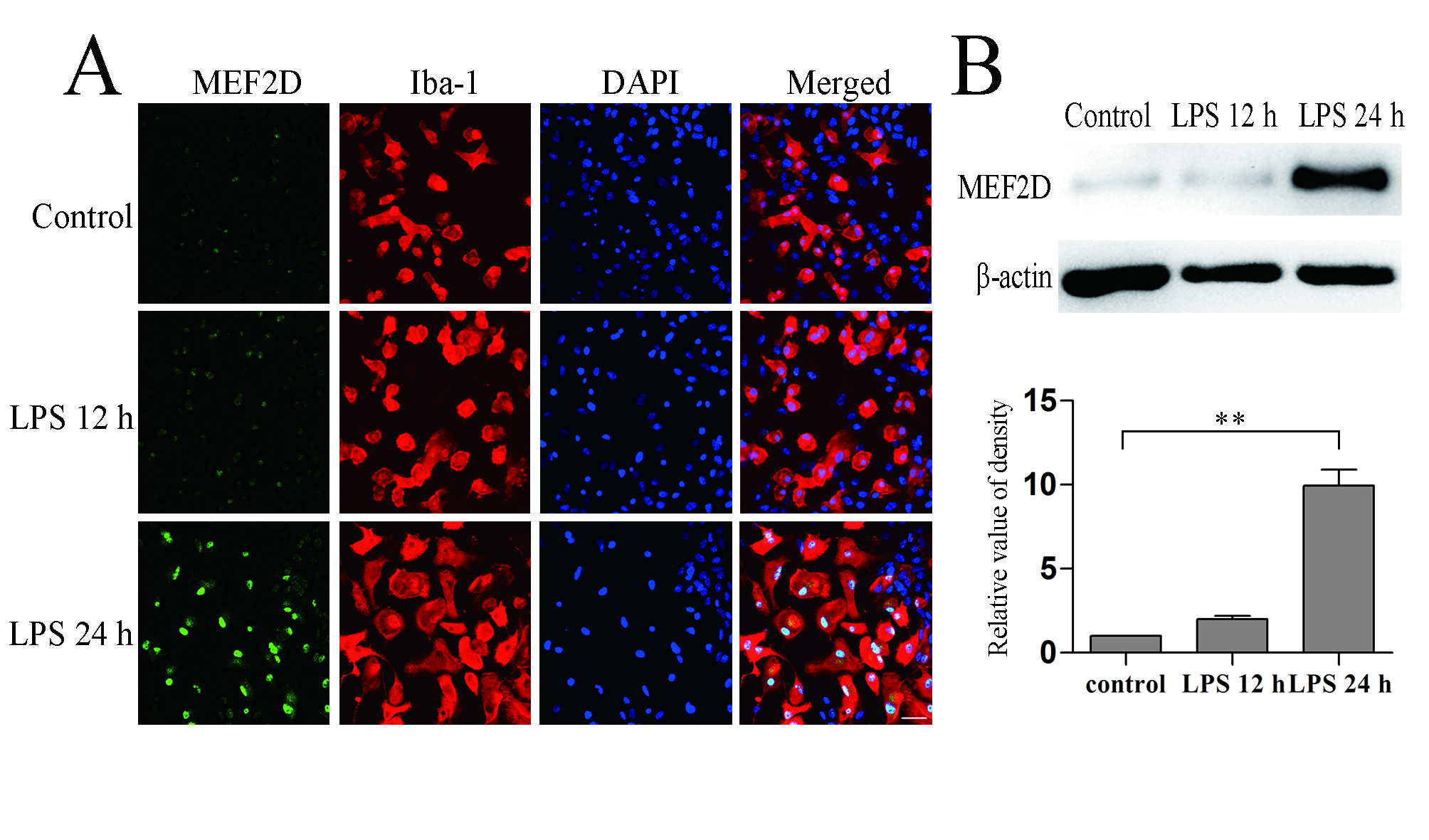

Supplement: Additional file 1: Figure S1. — Induction of MEF2D expression by LPS in mixed glial cells and purified primary microglia. (A) Mixed glial cells from rat were treated with 1.0 μg/ml LPS for the indicated time and analyzed by immunofluorescence (bar = 25 μm). (B) Purified primary microglia was analyzed by immunoblotting. Bottom graph shows the quantification of MEF2D. Data from three independent experiments were expressed as mean ± SEM and analyzed by one-way ANOVA (**P < 0.01). [file 12974_2015_258_MOESM1_ESM.tiff]
